# Neoadjuvant vascular-targeted photodynamic therapy improves survival and reduces recurrence and progression in a mouse model of urothelial cancer

**DOI:** 10.1038/s41598-021-84184-y

**Published:** 2021-03-01

**Authors:** Barak Rosenzweig, Renato B. Corradi, Sadna Budhu, Ricardo Alvim, Pedro Recabal, Stephen La Rosa, Alex Somma, Sebastien Monette, Avigdor Scherz, Kwanghee Kim, Jonathan A. Coleman

**Affiliations:** 1 Department of Surgery, Urology Service, Memorial Sloan Kettering Cancer Center, 1275 York Ave., New York, NY 10065 USA; 2 Department of Urology, Urologic-Oncology Service, The Chaim Sheba Medical Center, Affiliated with the Sackler School of Medicine, 5262080 Ramat Gan, Israel; 3 Department of Surgery, Sloan-Kettering Institute, Memorial Sloan Kettering Cancer Center, New York, NY USA; 4 Immunology Program, The Jedd Wolchok Lab, Memorial Sloan Kettering Cancer Center, New York, NY USA; 5 Laboratory of Comparative Pathology, Memorial Sloan Kettering Cancer Center, New York, NY USA; 6 Weill Cornell Medical College, New York, NY USA; 7 Department of Plant Sciences, Weizmann Institute of Science, Rehovot, Israel

**Keywords:** Cancer, Cancer therapy, Urological cancer

## Abstract

Locally advanced urothelial cancer has high recurrence and progression rates following surgical treatment. This highlights the need to develop neoadjuvant strategies that are both effective and well-tolerated. We hypothesized that neoadjuvant sub-ablative vascular-targeted photodynamic therapy (sbVTP), through its immunotherapeutic mechanism, would improve survival and reduce recurrence and progression in a murine model of urothelial cancer. After urothelial tumor implantation and 17 days before surgical resection, mice received neoadjuvant sbVTP (WST11; Tookad Soluble, Steba Biotech, France). Local and systemic response and survival served as measures of therapeutic efficacy, while immunohistochemistry and flow cytometry elucidated the immunotherapeutic mechanism. Data analysis included two-sided Kaplan–Meier, Mann–Whitney, and Fischer exact tests. Tumor volume was significantly smaller in sbVTP-treated animals than in controls (135 mm^3^ vs. 1222 mm^3^, *P* < 0.0001) on the day of surgery. Systemic progression was significantly lower in sbVTP-treated animals (l7% vs. 30%, *P* < 0.01). Both median progression-free survival and overall survival were significantly greater among animals that received sbVTP and surgery than among animals that received surgery alone (*P* < 0.05). Neoadjuvant-treated animals also demonstrated significantly lower local recurrence. Neoadjuvant sbVTP was associated with increased early antigen-presenting cells, and subsequent improvements in long-term memory and increases in effector and active T-cells in the spleen, lungs, and blood. In summary, neoadjuvant sbVTP delayed local and systemic progression, prolonged progression-free and overall survival, and reduced local recurrence, thereby demonstrating therapeutic efficacy through an immune-mediated response. These findings strongly support its evaluation in clinical trials.

## Introduction

Urothelial carcinomas (UC) can involve the lower (bladder and urethra) or upper (renal pelvis and ureters) urinary tract. The vast majority (90–95%) occur in the bladder. Urothelial bladder cancer (UBC) is the ninth most common cancer in the world^1^. The estimated deaths in the United States for UBC were 17,980 for 2020^2^.

Surgical treatment as sole modality can be curative, mainly for organ-confined, lymph-node negative disease. However, survival rates decrease dramatically for locally and regionally advanced disease^3^, even with aggressive surgical and medical interventions. Platinum-based neoadjuvant chemotherapy (NAC) for invasive UBC produces significant survival as well as disease-free survival benefit^4^ and is considered the most appropriate standard treatment for invasive UBC^5^. Although evidence supporting NAC in advanced upper-tract urothelial carcinoma (UTUC) is less well-developed, 45–60% of patients will progress following surgery, necessitating the use of chemotherapy^6^. Despite these data, NAC for UBC and UTUC has gained very limited clinical acceptance^7^, probably reflecting concerns over the toxicity associated with it.

A strong need therefore exists for developing innovative, less toxic, and more clinically acceptable neoadjuvant therapies for invasive urothelial carcinoma.

The importance of immunotherapy in the treatment of UC was first reported in 1971, when bacillus Calmette-Guerin **(**BCG) was used as a localized immunotherapy^8^. In the past few years, promising evidence has indicated an expanding role for systemic immunotherapy in urologic cancers including bladder cancer^9^. Although robust evidence supporting immunomodulating drugs in the adjuvant setting exists, there is a gap in knowledge about their role in the neoadjuvant scenario. Furthermore, because response to immune checkpoint inhibitors tends to be individualized and adverse events, while rare, tend to be potentially serious, immunomodulating drugs may be less appealing as a first-line neoadjuvant approach^10^.

Evidence supporting use of photodynamic therapy (PDT) for UC was introduced in the 1980s with long-term efficacy that suggested an immune-related response^11–13^. Later reports consistently supported the role of PDT in cancer treatment inducing an antitumor immune response^14–17^. Despite profound supporting evidence for PDT, limitations, such as prolonged phototoxicity, delayed clinical acceptance, prompting the development of better performing photosensitizing molecules^18^.

One of these better performing molecules, WST11 (Tookad Soluble, Steba Biotech, France), is a water-soluble, near-infrared-activated Pd-bacteriochlorophyll derivative^19–21^. Following intravenous administration, WST11 noncovalently binds to albumin and is sequestered within the circulation. Upon illumination within tissues, WST11 vascular-targeted photodynamic therapy (VTP) produces localized, soft-tissue ablation mediated by cytotoxic-reactive oxygen species and destroys malignant cells^22,23^. In contrast to former photodynamic therapies, VTP is confined to the vasculature of the tumor, arresting the tumor’s blood supply by rapid occlusion and causing profound tumor necrosis within 48 h^21^. WST11-VTP of tumor-bearing solid organs was found to be safe and effective in prostate cancer clinical trials^24–26^. In addition to its local effect, VTP has been shown to induce long-lasting systemic antitumor immunity including both cellular and humoral components^27,28^.

As locally applied ablation effects may be variable in advanced UC cancers, we sought to investigate the role for VTP as a neoadjuvant rather than primary therapy. Therefore, we utilized less optimal treatment conditions, or sub-ablative settings, in which tumors would be damaged but not completely destroyed. We used the murine bladder 49 (MB-49) cell line, known to metastasize spontaneously to lymph, spleen, and lungs. We transfected it with a luciferase-expressing gene to generate MB-49 luciferase-expressing (MB-49-luc) cell line.

## Results

### Experiment 1: Standalone sbVTP

The goal of this experiment was to determine the influence of sbVTP on overall survival (OS), metastasis-free survival (MFS), and tumor progression as compared to control and surgical resection.

**Figure 1:**
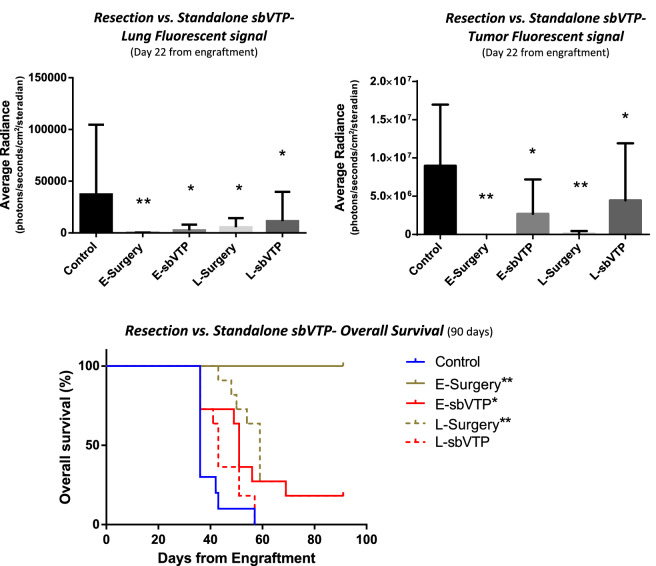
(Upper panel) Fluorescence signal from thorax and the primary tumor implant site in mice injected with MB-49 urothelial cancer cell line (*n* = 54). (Lower panel) Kaplan–Meier curves showing overall survival. Animals were treated with surgery or sbVTP, 2 or 3 weeks (early or late, respectively) after tumor injection, compared to control group (*n* = 11 per treated groups, *n* = 10 per control). *E-sbVTP* early sub-ablative vascular-targeted-photodynamic therapy, *E-Surgery* early tumor surgical resection, *L-sbVTP* late sub-ablative vascular-targeted-photodynamic therapy, *L-Surgery* late tumor surgical resection. *P < 0.05 as compared to control; **P < 0.0001 as compared to control.

sbVTP treatment led to reductions in tumor progression. By 22 days after tumor implantation, the early (2 weeks) sbVTP group showed tumor and lung fluorescence signals significantly lower than control (*P* < 0.05) but higher than the early surgery group (*P* < 0.0001) (Fig. 1 and Supplementary Table). Tumor radiance for the late (3 weeks) sbVTP group also demonstrated signals lower than control (*P* < 0.05).

Tumor size at day 21 similarly showed benefits for sbVTP treatment. The early and late sbVTP groups on this day had an average tumor size of 211 mm^3^ (95% CI, 50–372 mm^3^; *P* < 0.01) and 422 mm^3^ (95% CI, 163–682 mm^3^; *P* < 0.05) respectively, compared to with 769 mm^3^ (95% CI, 588–950 mm^3^) for control. Tumors could not be measured in surgically resected animals.

Early sbVTP demonstrated survival benefits compared to control but not to early surgery. OS and MFS were significantly longer in the early sbVTP group compared to control (*P* < 0.05), but shorter than in the early surgery group (*P* < 0.001). The late sbVTP group failed to show an advantage over control regarding these endpoints (Fig. 1). Median OS per group was as follows: control, 36 days; early surgery, undefined (did not reach below 50% survival for 90 days); early sbVTP, 51 days; late surgery, 59 days; and late sbVTP, 43 days. Median MFS per group was as follows: control, 22 days; early surgery, undefined (did not reach below 50% survival for 90 days); early sbVTP, 36 days; late surgery, 36 days; and late sbVTP, 29 days.

sbVTP possibly suggests long-term systemic immunity. When animals were rechallenged with tumor cells injection more than 100 days following treatment, no sbVTP (0/2) and only 1/13 of the surgically treated animals developed signs of local or systemic disease. In contrast, all control mice (*n* = 5) developed local tumors and metastasis, eventually succumbing to their disease (*P* < 0.05). A following experiment further describes additional group of long term sbVTP treated animals re-challenged with tumor cells (see ahead, flow cytometry—experiment 5).

### Experiment 2: Surgery timing following neoadjuvant sbVTP

The goal of this experiment was to investigate how surgery at different times affects OS.

sbVTP treatment, alone or in combination with surgical resection, demonstrated superior OS compared to control (*P* < 0.05). Surgical resection plus sbVTP was significantly superior to sbVTP alone if performed at days 17 and 24 from tumor engraftment (3 and 10 days following sbVTP, respectively; *P* < 0.001 and *P* < 0.05, respectively). However, for later time points representing advanced disease, i.e., 31 days from engraftment, the advantage of combining surgical resection with sbVTP over sbVTP alone was no longer significant (*P* = 0.1). Median OS per group was as follows: control, 43 days; sbVTP, 56 days; sbVTP + surgery at day 17, undefined (did not reach below 50% survival for 121 days); sbVTP + surgery at day 24, 69 days; and sbVTP + surgery at day 31, 66.5 days.

### Experiment 3: Systemic progression timeline–imaging validation

The goal of this experiment was to validate the time from engraftment with tumor cells to systemic progression.

The average day for positive lung fluorescence signal (that is, systemic progression) was 33.5 days (standard deviation, 5.5 days) from tumor engraftment, as calculated from the control groups of standalone sbVTP and surgery following neoadjuvant sbVTP (experiments 1 and 2). This timeline was confirmed by gross and microscopic analysis. Out of the 18 animals engrafted with tumors as part of Experiment 3, fourteen (78%) showed gross lung metastasis on day 33. Two additional animals were found to have lung metastases on microscopic analysis, bringing the total to 16 (89%).

### Experiment 4: Neoadjuvant sbVTP

The goal of this experiment was to define the effects of neoadjuvant sbVTP on OS, progression-free survival (PFS), tumor volume at surgery, and local recurrence as compared to control. sbVTP and surgical resection were performed 14 and 31 days following implantation of tumor cells, respectively with the intent of treating micrometastatic disease. Neoadjuvant treatment with sbVTP produced improvements in OS and PFS, reduced tumor volume at surgery, and decreased local recurrence following it. Average tumor volume at the day of sbVTP was 70 mm^3^ [standard error (SE), 4.1; 95% CI, 60–80 mm^3^]. At day of surgery tumor volume for sbVTP-treated animals (i.e., sbVTP and sbVTP + surgery at day 31[VTPS]) was 135 mm^3^ (SE, 35; 95% CI, 66–204 mm^3^) and for non-sbVTP-treated animals (i.e., control and surgery only) was 1222 mm^3^ (SE, 125; 95% CI, 976–1468 mm^3^) (*P* < 0.0001). Animals treated with sbVTP had a higher rate of tumor regression and a lower rate of systemic progression on the day of surgery (Table 1).

**Table 1:** Tumor regression and systemic progression by treatment group prior to surgical resection.

Neoadjuvant treatment	Group	Tumor regression	Tumor regression (overall, %)	Death	Lung metastasis	Systemic progression (overall, %)
None	Control	0	0/29 (0)*	1	1	9/30 (30)*
	Surgery	0		0	7	
sbVTP	sbVTP	7	12/45 (27)*	0	2	3/45 (7)*
	VTPS	5		0	1	

*sbVTP* sub-ablative vascular-targeted photodynamic therapy, *VTPS* sub-ablative vascular-targeted photodynamic therapy followed by surgical resection of tumor. One control animal was censored from the tumor regression analysis due to death prior to surgery day.

*P < 0.05.

**Figure 2:**
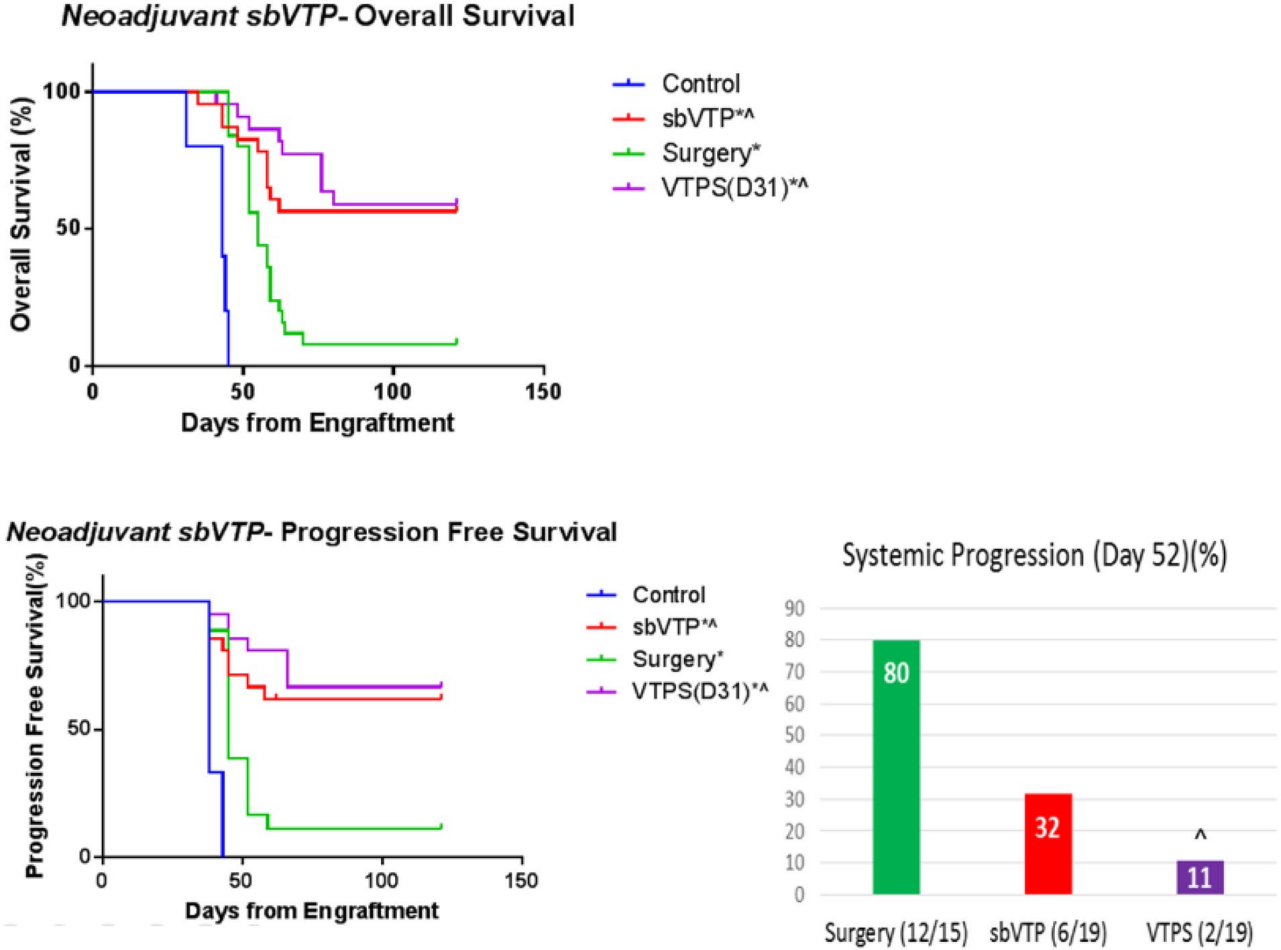
Kaplan–Meier analysis for overall survival and progression-free survival. Surgical resection was performed on day 31 for relevant groups (not illustrated). Control (*n* = 5); *sbVTP* sub-ablative vascular targeted photodynamic therapy (*n* = 23); Surgery (*n* = 25); *VTPS(D31)* sub-ablative vascular-targeted photodynamic therapy followed by surgical resection of tumor 31 days from engraftment (*n* = 22). Two and 3 animals were censored from the analysis in the sbVTP and VTPS groups, respectively, due to peri-procedure death. *P < 0.05 versus control; ^*P* < 0.05 versus Surgery group. *Note* The improved survival presented for sbVTP at 2 weeks as compared to Fig. 1 is attributed to the different sizes of the treated tumors (see “Discussion” section).

OS and PFS were significantly longer for VTPS as compared to the control and surgery only groups (Fig. 2, upper panel). Median OS per group was as follows: control, 43 days; sbVTP, undefined (did not reach below 50% survival for 121 days); surgery only, 55 days; and VTPS, undefined (did not reach below 50% survival for 121 days). Median PFS per group was as follows: control, 38 days; sbVTP, undefined (did not reach below 50% survival for 121 days); surgery only, 45 days; and VTPS, undefined (did not reach below 50% survival for 121 days).

Survival curves analysis in the short term following surgery (day 52 from engraftment) showed PFS for the VTPS group to be significantly longer than that for the sbVTP and surgery-only groups (*P* < 0.05) (Fig. 2, lower right panel).

**Figure 3:**
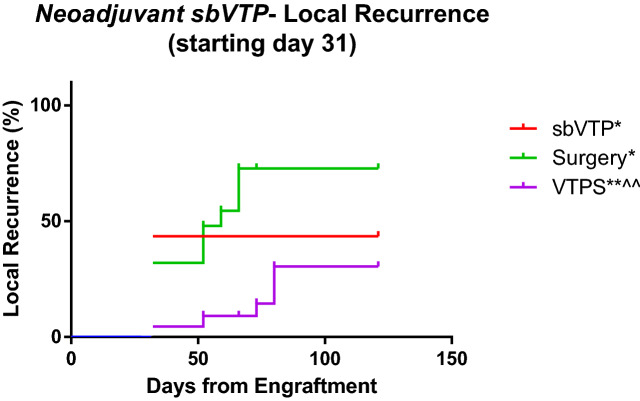
Kaplan–Meier analysis for local recurrence 121 days from tumor engraftment. Analysis started 31 days following tumor engraftment (day of tumor surgical resection for relevant groups). *sbVTP* sub-ablative vascular-targeted photodynamic therapy (*n* = 23), *VTPS* sub-ablative vascular-targeted photodynamic therapy followed by surgical resection of tumor (*n* = 22); Surgery (*n* = 25). *P < 0.05 versus control; **P < 0.01 versus control; ^^*P* < 0.001 versus Surgery group. Control (*n* = 5) animals were omitted from figure as no “recurrence”.

Time to local recurrence was significantly longer for VTPS as compared to control and surgery-only groups (Fig. 3). Median local recurrence per group was as follows: control, 31 days; sbVTP, undefined (did not reach above 50% recurrence for 121 days); surgery only, 59 days; and VTPS, undefined (did not reach above 50% recurrence for 121 days).

### Experiment 5: Flow cytometry and IHC

The goal of this experiment was to elucidate the mechanism of sbVTP and its long-term effect. sbVTP induced a long-term immune-mediated response based on our analysis of resected tumors, lungs, spleens, and blood using IHC and flow cytometry.

**Figure 4:**
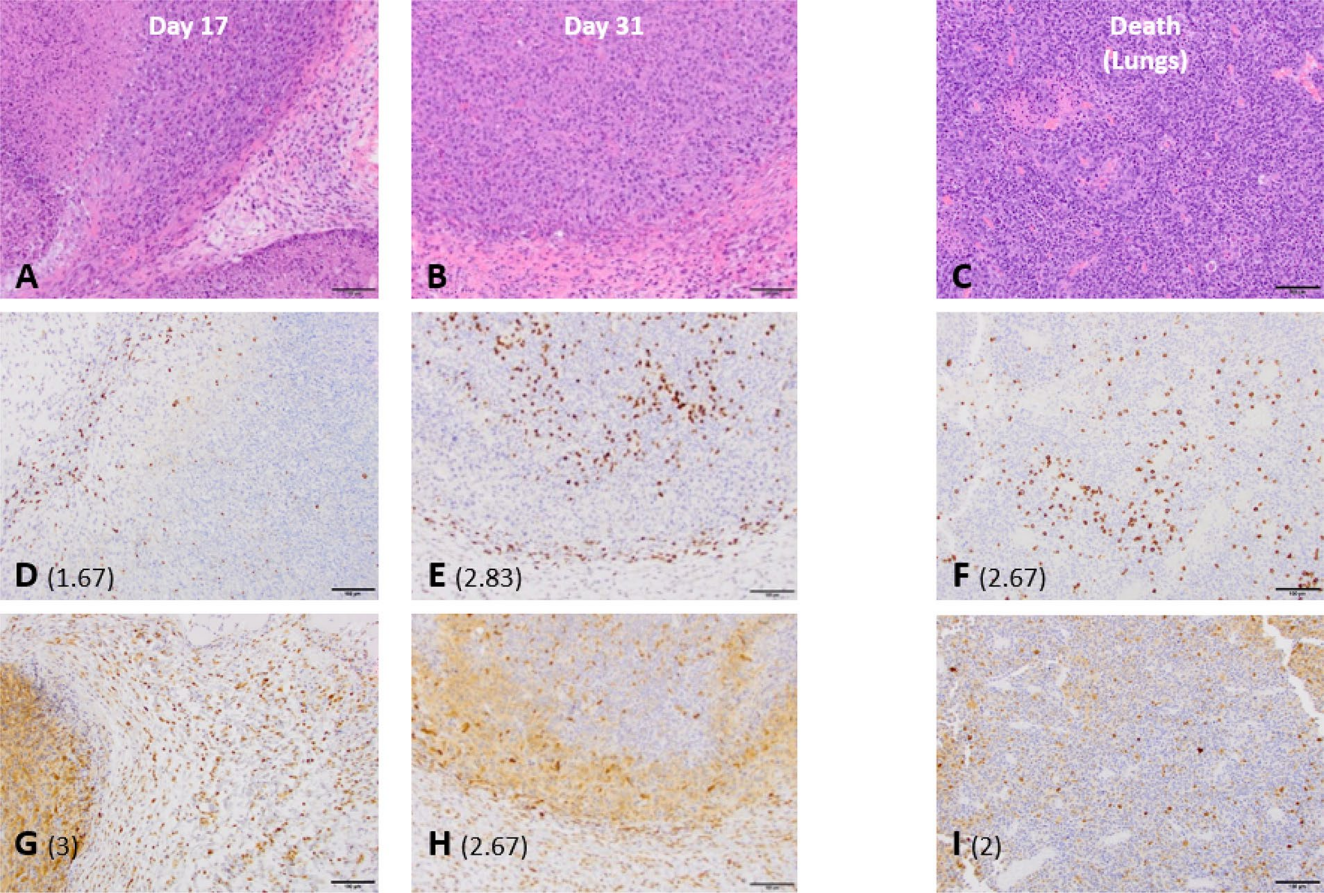
Tumor and lung histology analysis showing evolving immune reaction at tumor site following sub-ablative vascular-targeted photodynamic therapy. Semi-quantitative score (shown in parentheses) indicates the number of positive cells infiltrating tumor and peri-tumoral tissues, on a scale of 0 (no positive cells) to 4 (large number of positive cells). CD3 + is shown in (**D**–**F**), and Mac2 in (**G**–**I**), with both cell types observed in the same region. (**A**,**B**) Display results of hematoxylin and eosin (H&E) stain, showing skin, subcutis, and muscle with subcutaneous neoplasm. Note the chronic-active inflammation of tumor and adjacent tissues. (**D**,**E**) display results of CD3 + stain, illustrating time-dependent accumulation of T-cells. (**G**,**H**) display results of Mac2 (galectin 3) stain, illustrating initial accumulation of macrophages and dendritic cells followed by gradual decline. Lungs showed metastatic foci on H&E (**C**), aggregation of T-cells (CD3 + stain, **F**), and macrophages and dendritic aggregation (**I**).

Tumor and lung histology analysis showed an evolving immune reaction at tumor site following sbVTP (Fig. 4). Chronic-active inflammation of tumor and adjacent tissues at days 17, 24, and 31 was shown by hematoxylin and eosin (H&E) staining. The lowest daily average tumor CD3 + (T-cells) score was 1.67 three days following sbVTP (i.e., day 17 from engraftment). This nadir was followed by gradual increases on day 24 (2.67) and day 31 (2.83). Average lung CD3 + score at death was 2.63. The average tumor Mac2 (macrophages and dendritic cells) score showed an opposite trend, reaching 3, its highest point, 3 days following sbVTP and then decreasing to 2.67 on day 24 and day 31. The average lung Mac2 score reached its lowest point (2) at death (Fig. 4). Surgery-only samples of tumor (day 31) and lungs (at death) showed similar directional trends (i.e., tumor score rising and lung score falling) although to a lesser degree (not shown).

**Figure 5:**
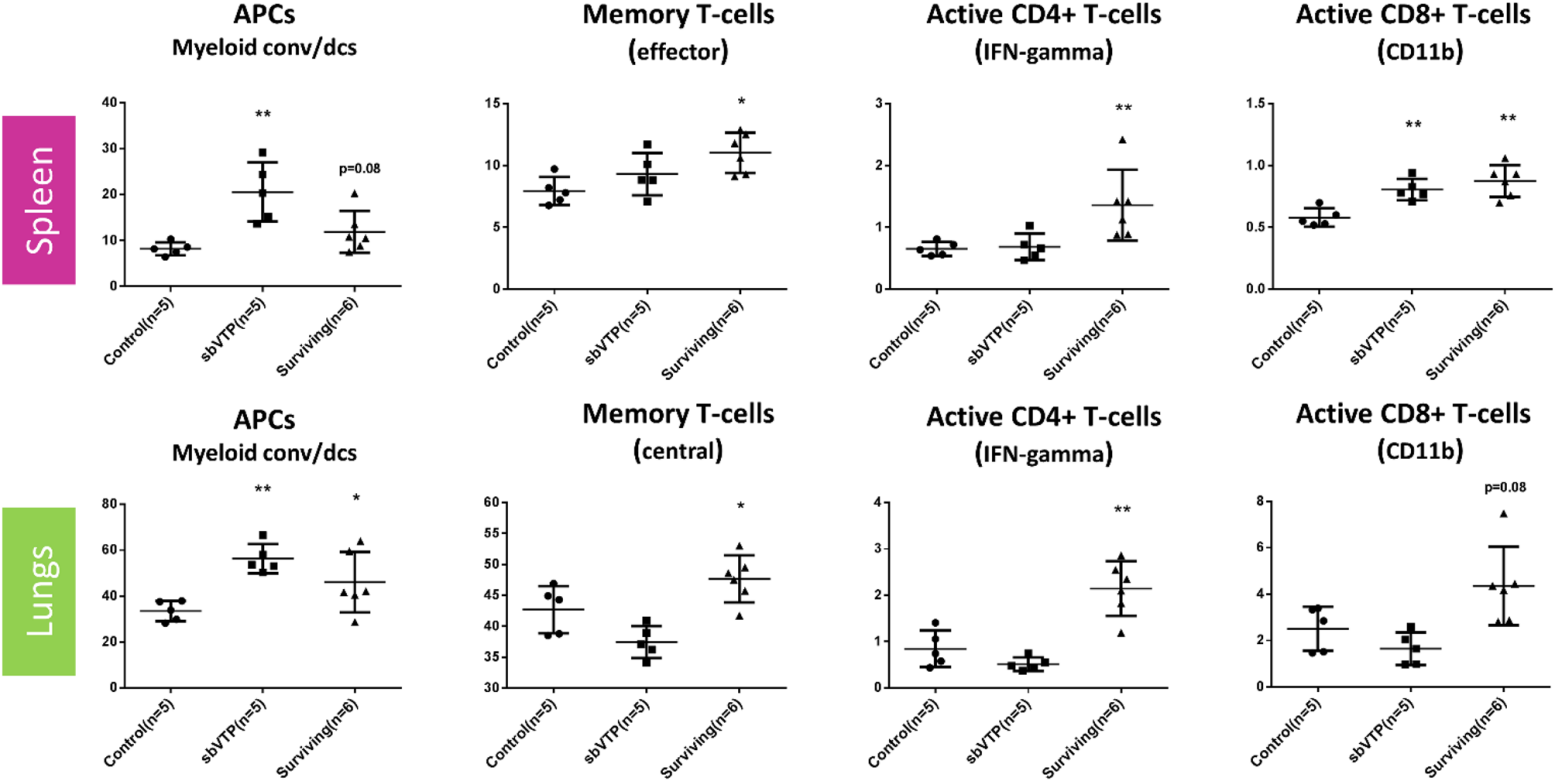
Flow data: cells’ sub-population percentage by organ. Left to right: first column represents antigen-presenting cells (APCs), second column represents memory T-cells (central/effector), third column represents active CD4 + T-cells as indicated by IFN-gamma positivity, and fourth column represents active CD8 + T-cells as indicated by IFN-gamma and CD11B positivity. CD45 +, CD4 +, Foxp3-were used to evaluate CD4 T effector cells. CD62L + CD44 + were used to evaluate central memory T cells and CD62L-CD44 + to evaluate effector or effector memory T cells. *APCs* antigen-presenting cells, *conv* conventional, *dcs* dendritic cells, *IFN* interferon, *sbVTP* sub-ablative vascular-targeted photodynamic therapy, *surviving* surviving animals. *P ≤ 0.05; **P < 0.01, compared to control.

sbVTP was associated with an early increase in antigen-presenting cells (APCs) as well as long-lasting immune responses. When immune cells were analyzed using flow cytometry in spleen, lungs, and blood to assess systemic response (Fig. 5; supplementary Fig. 1), APCs were significantly higher 3 days following sbVTP throughout all three foci. Surviving animals showed increased CD8 + T-cells in the spleens and increased memory T-cells and effector T-cells in all foci. Blood analysis of surviving animals found that both CD4 + and CD8 + active T-cells trended toward higher levels. Following tumor rechallenge to these animals, active CD4 + T-cells in blood were significantly higher.

## Discussion

The five experiments in our study demonstrate that partial tumor ablation with sbVTP is an effective neoadjuvant treatment for urothelial cancer.

sbVTP reduces systemic progression and improves survival compared to control at early time points (Experiment 1). At late time points, representing advanced disease, sbVTP with or without surgery provided long-term benefit compared to surgical resection alone (Experiments 2 & 4). Although sbVTP advantage is shown in both experiments, we report differences in overall survival of animals treated by sbVTP only (Figs. 1, 2). We attribute this difference to tumor size which was larger in day of sbVTP treatment in experiments 1 and 2 (avg. ~ 200 cc) versus experiment 4 (avg. ~ 70 cc), and hence may have influenced sbVTP outcomes. The fact that sbVTP reduces systemic progression and improves survival suggests it to potentially reduce or eliminate seeding of micro-metastases and systemic progression while local tumor resection cannot (Fig. 2; Table 1).

These findings may help explain published data reporting a higher failure rate for surgical resection of UBC^3^ and UTUC^6^ in advanced disease, most probably caused by concealed systemic disease at the time of surgical resection. In our UC mouse model, we found day 33 to represent the onset of clinically evident metastatic disease. From this, we can infer that advanced disease characterized by concealed micro-metastases is highly probable at day 31. Indeed, our Experiment 4 showed that sbVTP treatment *before* surgery at day 31 was more effective than surgery alone at that day. This suggests that the cause of the higher failure rate for surgical treatment of advanced disease may be the existence of concealed micro-metastases. Therefore, in humans, we believe the optimal time for treating micro-metastases with sbVTP is *before* these metastases become clinically evident—that is, as neoadjuvant treatment. Supporting this recommendation, bladder cancer regression following neoadjuvant treatment has been correlated with improved recurrence-free survival^29^ and OS^30^. Similarly, our model showed that animals treated with sbVTP prior to surgical resection have higher rates of tumor regression and lower rates of systemic progression prior surgery (Table 1) translated in to longer PFS and OS.

Our findings suggest that sbVTP produces a long-term systemic immune response. Tumor histology analysis (Experiment 5) showed chronic-active inflammation of tumor and adjacent tissues as abundant macrophages and T-cells infiltrated them (Fig. 4). Rechallenging mice more than 100 days after sbVTP resulted in no uptake of tumor grafts. Systemic progression was delayed following sbVTP (Table 1). These findings confirm published literature defining immune response as one of the main mechanisms by which PDT destroys tumor cells and initiates the long-term systemic immune response that follows^14,17,28,31,32^.

In the long term, sbVTP produced superior PFS compared to all other treatments. In the early weeks, the VTPS group showed a significant advantage in PFS compared to the surgery-only group and the sbVTP-only group (Fig. 2). The VTPS group’s advantage over the sbVTP-only group, however, later became non-significant, and in the long-term both groups receiving sbVTP (VTPS and sbVTP-only) demonstrated significantly longer PFS. A possible explanation for this phenomenon may be suggested by the immunoediting theory^33^. sbVTP may be sculpting the immunogenicity of the tumor cells, tilting the host-tumor immune balance toward an equilibrium state. Indeed, PDT may emit a powerful attracting signal for immune cells that can be engaged in additional eradication of disseminated and/or metastatic lesions of the same cancer^15,34^. VTP using WST11 also initiated a systemic immune response and established a prolonged immunity in animal cancer models in previous studies^27,28^. Our study found evidence of this immune-response mechanism by using Mac2, one marker of macrophage maturation in mice^35,36^. Using Mac2 tagging, we found an initial proliferation of dendritic cells and macrophages early after sbVTP and a subsequent reduction in their magnitude. Mac2 and CD3 + stained cells both demonstrated the ability to infiltrate tumor and peritumoral tissues (Fig. 4). Massive invasion of cancer tissue by activated myeloid cells following PDT enables tumor antigens to present and subsequently activate lymphoid cells, leading to tumor-specific immunity^14,17,34^. The combination of macrophages and T-cells is reportedly essential to maintaining long-term control of PDT-treated tumors^32^. This mechanism appears to explain why sbVTP leads to longer PFS and OS.

Our study provided further details about this important mechanism. Our flow analysis showed APCs increasing in the early phase after sbVTP throughout all three systemic foci (spleen, lungs, and peripheral blood) (Fig. 5 and supplementary Fig. 1). Correspondingly, surviving animals showed an increase in memory T-cells and effector T-cells at all foci. These data illustrate the proposed “crosstalk” between antigen presentation, T-cells, and tumor cells as suggested by the immunoediting theory^33^. This crosstalk initiates the adaptive immune response that takes place long after sbVTP is administered. Identification of active T-cells in all foci further supports that actual tumor elimination/equilibrium is occurring in surviving animals. Finding the populations of CD8 + T-cells to be significantly higher in surviving animals suggests their pivotal role in the antitumor, immune-mediated, sbVTP effect. Indeed, Murphy et al. suggested CD8 + T-cells and IFN-gamma promote antitumor responses in advanced metastatic cancer^37^. The higher levels of active CD4 + T-cells in spleen and lungs, which were harvested long after treatment, further suggests that active antitumor immune reaction continues well after sbVTP^38,39^.

sbVTP can potentially reduce metachronous UBC following UBC and UTUC. Under current treatment regimens, some 15–50% of patients with a UTUC will subsequently develop a metachronous UBC. In most cases, UBC arises in the first 2 years after UTUC management. However, the post-UTUC risk of UBC is lifelong and repeated episodes are common, as reflected by the strict and frequent follow-up guidelines recommended by the NCCN^5,40^. Immune-modulating drugs such as BCG have been shown to delay UC recurrence among other benefits^41–43^, and intravesical PDT was reported to delay bladder recurrence^44^. On top of our findings that sbVTP improves survival and regression rates, our finding that sbVTP has a long-lasting systemic effect can potentially benefit UC patients by lowering recurrence rates.

Drug toxicity—in particular, renal impairment—is the major impediment to perioperative chemotherapy in patients with bladder cancer^29^. As many as 50% of patients have renal impairment, and approximately one third have other comorbidities that may preclude cisplatin-based treatment. VTP using WST11 appears to be an acceptable form of treatment for at least some and perhaps all of these patients, based on our knowledge to date. Although our experiments were not designed to assess the toxicity of sbVTP, we observed nothing that would suggest any toxicity issues, supporting recent studies. When sbVTP was tried on normal swine model tissue, as our group reported before, it preserved critical organ structures and bystander blood vessels within solid organs, as well as functionally preserving creatinine value within a normal physiological range^45,46^. Notably, VTP using WST11 is reported to be well-tolerated in men with prostate cancer^47^. VTP may enhance the neoadjuvant arsenal by offering a similar, if not superior, long-term effect at a considerably lower toxicity than existing treatments.

UTUC is oftentimes diagnosed using an endoluminal or percutaneous approach. Since a similar surgical approach can be used for VTP^46^, we believe that VTP treatment is simple and convenient enough to use even at the time of biopsy. This approach could enable the urologist to locally apply potent systemic treatment at the time of diagnosis, proceeding to curative surgical resection without any delay. As mentioned above, in contrast to former photodynamic therapies, VTP is confined to the vasculature of the tumor, arresting the tumor’s blood supply by rapid occlusion and causing profound tumor necrosis within 48 h, a reaction which is strongly immunogenic compared with the apoptotic cell death with the other reagents^21^. Considering the benefits of neoadjuvant photodynamic therapy, we suggest alternatives such as Visudyne and 5-aminolevulinic acid, previously described to effect urothelial cancer^48–51^, to be tested in this setting as well.

Our study has limitations typical to the use of animal models to approximate the treatment conditions and outcomes that might be applicable in humans. Future usage of orthotopic bladder cancer mouse model may better simulate urothelial cancer pathophysiology and validate our findings. Engraftment of luciferase-expressing tumor cells via an intra-urethral approach, followed by spontaneous systemic metastasis of these tumor cells, will probably create a better model to investigate our results. However, we are currently unaware of such stable model to exist. In addition, our study has some limitations applicable to the particular design of it. We used treatment parameters that were below the level of complete ablation, which introduces variability in the treatment effects to the primary tumor. This is reflected in the range of responses at the local tumor site. Our focus, however, was primarily on the systemic effects associated with partial treatment as well as on the effects in the tumor microenvironment under these conditions. Although optimal conditions would be expected to improve VTP therapy outcomes locally, our technique may better simulate the clinical scenario in which, for example, complete endoscopic treatment to an upper urinary tract tumor might not be feasible. Furthermore, the immune-modulating effect of sbVTP as compared to VTP is yet to be elucidated. Another limitation is that in our testing for mechanism, we compared younger mice to surviving animals who were 3 months older (Experiment 5). Although protective immunity may decline with age, mice are not considered “old” until they are over 18 months^52^, and we therefore believe that an age difference between 1 and 4 months, as in our study, is unlikely to confound our findings.

In summary, vascular-targeted photodynamic therapy, performed under sub-ablative parameters, demonstrates long-lasting therapeutic efficacy in a mouse model of urothelial cancer. Applying this treatment neoadjuvantly delays local and systemic progression prior to surgery, prolongs both PFS and OS, and reduces local recurrence. This therapy induces an early increase in antigen-presenting cells, followed by increases in long-term memory cells, effector cells, and active T-cells, establishing its immune-mediated mechanism. These findings provide a strong rationale for evaluating this therapy in clinical trials of locally advanced urothelial cancer.

## Materials and methods

Our experiments applied a syngeneic non-orthotopic model. Cell culture, vascular targeted photodynamic therapy (VTP), imaging and tissue analysis were based on prior work by our group^53,54^.

### Cell culture

In brief, murine bladder 49 (MB-49) is a carcinogen-induced urothelial cell carcinoma derived from male C57BL/6 mice (Taconic Farms, New York, NY, USA). The MB-49 cells were cultured in Dulbecco's modified eagle medium supplemented with 10% fetal bovine serum, 1% penicillin/streptomycin, and 0.1% sodium pyruvate. Murine stem cell virus puromycin–luciferase–GFP was transfected into GP2-293 pantropic retroviral packaging cells (BD Biosciences, San Jose, CA, USA) using lipofectamin 2000 (Invitrogen, Grand Island, NY, USA), and the collected retrovirus was used to infect cells from the MB-49 mouse-bladder cancer cell line. Infected cells were selected with 0.5 μg/mL puromycin (Invitrogen) and the surviving pool of cells was designated as MB-49-luc. Infection was carried out in the presence of 6 μg/mL polybrene (Sigma, St. Louis, MO, USA). Prior to injection, MB-49-luc cells were washed and re-suspended in phosphate-buffered saline (PBS) pH 7.4 and viable cells were counted using Trypan blue exclusion and a hemocytometer. Tumor cells were subcutaneously injected in animals’ right flank. For all experiments, the same number of MB-49-luc cells was injected (50 K).

### Tumor measurement

The size of the primary tumor was assessed by caliper and validated by bioluminescence signals. The progression of lung metastases was monitored by luminescent imaging and validated by immunohistochemistry (see Experiment 3 below).

### Animals

The study was performed using 7–8-week-old male C57BL/6 mice (Taconic Farms). Animals were housed in a light-controlled room with a 12:12-h light–dark cycle and allowed access to water and food ad libitum.

### Sub-ablative vascular-targeted photodynamic therapy (sbVTP)

Lyophilized WST11 was reconstituted with sterile 5% dextrose water under light-protected conditions and filtered through 0.22-μm disc filter. Infusion of weight-based WST11 at 9 mg/kg was administered via tail vein injection for 5 min. Two min after completing the drug injection, laser illumination was provided by a 753-nm medical diode laser (Biolitec, East Longmeadow, MA, USA) with front-face fiber optics. The light beam was adjusted to cover the same area regardless of fluency assuming an average tumor size for the different treated animals at 2 weeks post grafting. Light was delivered for 10 min at a fluency of ~ 122 mW/cm^2^. Under these conditions an increased number of animals showed tumor relapse starting at the treated tumors margin. This observation reflects on the Gaussian shape of the laser beam and the threshold dependence of the VTP treatment efficacy. Due to the Gaussian shape of the applied beam the margin of illumination is the first to drop below that threshold when reducing the light fluency from 150 to 122 mW/cm^2^ allowing for tumor relapse at the tumor margin. Complementary, slight increase of the tumor margin further decrease the cure rate. Accordingly, under the treatment conditions and the illuminated area size, we considered the amount of energy delivered to the tumor at 122 mW/cm^2^ as sub-ablative compared to former studies by our group^53^.

### sbVTP and surgical resection

Adequate anesthesia with a ketamine–xylazine cocktail (150 mg/kg ketamine, 10 mg/kg xylazine) and isoflurane, was administered prior to sbVTP treatments. For surgical resection of tumor animals were anesthetized by volatile fluorocarbon isoflurane (2.5%) administered with a precision vaporizer in an induction chamber, followed by use of a nose cone. After a suitable anesthetic plane (no response to stimulation) was attained, the animal was placed in position for mass removal on a surgical tray with heat support provided by a SnuggleSafe (Pet Supply Imports, South Holland, IL, USA). An injection of 2 mg/kg of meloxicam was given for pre-emptive analgesia immediately after the animal was anesthetized. Skin was then shaved and prepped for surgery. Tumors were resected *en bloc* with overlying skin and surrounding tissues as required to achieve zero margins. Hemostasis was achieved by application of local pressure. Skin was re-approximated using 9 mm surgical clips (AutoClips, Braintree Scientific, Braintree, MA, USA). Animal care before and during the experimental procedures was conducted in accordance with the policies of the National Institutes of Health Guidelines for the Care and Use of Laboratory Animals. All protocols received prior approval by the Institutional Animal Care and Use Committee.

### Luminescence imaging

Imaging was performed with a highly sensitive, cooled, charge-coupled device camera mounted in a light-tight specimen box (IVIS, Xenogen, Alameda, CA, USA). The acquisition and analysis software Living Image (Xenogen) were used for imaging and quantification of tumor progression. Following anesthesia with 1–2.5% isoflurane, mice were retro-orbitally injected with d-luciferin (PerkinElmer, Waltham, MA, USA) at a dose of 3 mg per mouse. Mice were then placed inside the light-tight camera box with continuous exposure to 1–2% isoflurane. Imaging time ranged from 1 s to 2 min. The level of light emitted from the tumors or metastatic foci was detected by the IVIS camera system, integrated, and displayed. Regions of interest from displayed images were designated around the tumor and lung sites and quantified using the software. Absolute signal measurement was corrected for background bioluminescence in vivo.

### Tissue fixation and histological evaluation

Tumors and lungs were harvested, preserved, and analyzed. During lungs processing, the trachea was identified and injected with approximately 0.5 to 1 mL of India ink stain (Boston BioProducts, Ashland, MA, USA) and the lungs were inflated adequately. Lungs were then removed *en bloc*, excluding all other tissues, and placed in Fekete’s solution for 24 h^55^. After collection, mice tumors and lungs were fixed in 10% neutral buffered formalin, processed in alcohol and xylene, paraffin embedded, sectioned at 5-µm thickness, and then stained with hematoxylin and eosin, CD3 + , and Mac2; immunohistochemistry (IHC) for CD3 was performed on a Leica Bond RX automated staining platform (Leica Biosystems, Buffalo Grove, IL, USA). Following heat-induced epitope retrieval (HIER) at pH 9.0, the primary antibody (rabbit monoclonal, catalog #ab16669, Abcam, Cambridge, UK) was applied at a concentration of 1:100 and was followed by application of a polymer detection system (DS9800, Novocastra Bond Polymer Refine Detection, Leica Biosystems). For IHC for Mac2, the antibody CL8942B (Cedarlane, Burlington, NC, USA) applied at a concentration of 1:100 following HIER in a pH 6.0 buffer; Mac2 staining was performed manually with an avidin–biotin detection system (Vectastain ABC Elite Kit, Vector Laboratories, Burlingame, CA, USA). We used a semiquantitative score to indicate the number of positive CD3 + and Mac2 cells infiltrating tumor and peritumoral tissues, on a scale of 0 (no positive cells) to 4 (large number of positive cells). Analysis was performed by a board-certified veterinary pathologist.

### Fluorescence-activated staining for surface antigens and intracellular proteins

Cell suspensions were incubated in Fc-block (CD16/32 antibodies, BD Biosciences) for 20 min on ice in PBS + 0.5% bovine serum albumin + 2 mm ethylenediaminetetraacetic acid [fluorescence-activated cell sorting (FACS) buffer] prior to surface staining. Samples were incubated with fluorophore conjugated CD4, CD8, CD25, CD62L, CD44, CD45, CD11c, Ly6G, Ly6C, MHC II, CD86, CD11b, and TGFβ for 20–30 min and then washed three times with FACS buffer. Foxp3 Staining Kit (eBioscience, San Diego, CA, USA) was used for intracellular staining of Foxp3, Ki67, and Granzyme B. Dead cell exclusion was done using the Fixable Viability Dye eFluor 506 (eBioscience). Samples were acquired on 12-color LSRII cytometer and analyzed using FlowJo software (Tree Star, Ashland, OR, USA).

### Experiments

Using animals’ survival, tumor measurements, luminescence imaging, immunohistochemistry, and flow cytometry, five experiments were conducted.

#### Experiment 1: Standalone sbVTP

The goal of this experiment was to determine the influence of sbVTP on OS, metastasis-free survival (MFS), and tumor progression as compared to control and surgical resection. Fifty-four animals were allocated into five groups based on tumor equivalent luciferase signal prior to treatment: control (no treatment, *n* = 10); early surgical resection (*n* = 11); early sbVTP (*n* = 11); late surgical resection (*n* = 11); and late sbVTP (*n* = 11). For sbVTP treatment, WST11 was administered intravenously as previously described. Surgery and sbVTP were performed at 2 weeks (“early”) or 3 weeks (“late”) after the implantation of tumor cells. Surgical margins were assessed by histology. All groups were examined with weekly luminescent imaging to determine MFS and the extent of progression. OS data was calculated.

A second phase of this experiment was designed to assess for development of systemic immunity following sbVTP and to measure time from engraftment to systemic progression using luminescent imaging. In this phase, all surviving animals (11 from early surgery, 2 from late surgery, and 2 from early sbVTP) were re-challenged 122 days after first tumor engraftment and over 100 days following any form of treatment. Animals were injected with MB-49-luc cells on the contralateral flank as previously described. Endpoints were the same clinical outcomes as before (OS, MFS, and local recurrence). Five treatment-naïve mice served as control.

#### Experiment 2: Surgery timing following neoadjuvant sbVTP

The goal of this experiment was to investigate how surgery at different times affects OS. In repeated sets of experiments, animals were allocated into five groups: control (no treatment; *n* = 5), sbVTP alone (*n* = 26), and three groups with the combination of sbVTP and surgery at different time points. The latter groups received sbVTP 14 days after the tumor cells were implanted, followed by surgical resection of the tumor at three time points: 17 days after implantation of tumor cells (*n* = 25), 24 days after implantation (*n* = 22), and 31 days after implantation (*n* = 18). All groups were examined with weekly luminescent imaging to determine time to progression (for Experiment 3) and the extent of progression (data not reported). OS data was calculated. Resected tumor tissue was preserved for further analysis, and sample animals’ lungs were harvested and preserved after the animals had succumbed to their disease.

#### Experiment 3: Systemic progression timeline–imaging validation

The goal of this experiment was to validate the time from engraftment with tumor cells to systemic progression. The average day of positive lung luminescence (a marker for systemic progression) was calculated from the control groups of the two previous experiments. Eighteen treatment-naive animals were engrafted with tumor cells. On the day that was calculated for positive lung fluorescence, animals were euthanized to permit physical validation of systemic progression. Gross metastases were assessed using India ink stain (Boston BioProducts, Ashland, MA, USA). Grossly negative or inconclusive lungs were assessed for micro-metastases using immunohistochemistry (IHC).

#### Experiment 4: Neoadjuvant sbVTP

The goal of this experiment was to define the effects of neoadjuvant sbVTP on OS, progression-free survival (PFS), tumor volume at surgery, and local recurrence as compared to control. Eighty animals were allocated into four groups: control (no treatment, *n* = 5); sbVTP (*n* = 25); surgical resection (*n* = 25); and the combination of sbVTP and surgical resection (VTPS, *n* = 25). sbVTP and surgical resection were performed 14 and 31 days following implantation of tumor cells, respectively. Progression was defined as positive lung signal determined by an in vivo imaging system or death. Resected tumor tissue was preserved for further analysis. Animal lungs were harvested and preserved at the time of necropsy after animals expired.

#### Experiment 5: Flow cytometry and immunohistochemistry

The goal of this experiment was to elucidate the mechanism of sbVTP and its long-term effect. Using immunohistochemistry, we analyzed tumor tissue collected on days 17 and 31 post-engraftment and lungs collected at time of death in experiments 2 and 4. In addition, we used naïve mice and surviving animals from Experiment 4. Animals were allocated into four groups: control (naïve mice, no treatment, *n* = 5); sbVTP (naïve mice treated with sbVTP, *n* = 5); long-term surviving animals (*n* = 6); and long-term surviving re-challenged animals (*n* = 3). Control, sbVTP, and long-term re-challenged animals were engrafted with tumor cells as previously described. sbVTP treatment took place on the fourteenth day after tumor engraftment for the sbVTP group. Mice were sacrificed 3 days after sbVTP (17 days after tumor engraftment for control and long-term re-challenged animals). Long-term surviving animals were sacrificed with no additional intervention; spleens, lungs, and blood were collected for flow analysis. We used intracellular interferon (IFN)-gamma + and CD11b + as markers for T-cell activity as suggested by Cristensen et al.^56^ and Fiorentini et al.^57^.

### Statistical analysis

Kaplan–Meier plots were used to analyze OS, PFS, and local recurrence. Comparative analysis of different groups was performed using the Mann–Whitney *U* test. Fisher’s exact test was used for Table 1. Statistical evaluations were performed using the GraphPad Prism software (GraphPad Software, La Jolla, CA, USA).

## Supplementary Information

Supplementary Information.

## Abbreviations

sbVTP: Sub-ablative vascular-targeted photodynamic therapy
VTP: Vascular-targeted photodynamic therapy
UC: Urothelial carcinomas
UBC: Urothelial bladder cancer
NAC: Neoadjuvant chemotherapy
UTUC: Upper-tract urothelial carcinoma
BCG: Bacillus Calmette–Guerin
PDT: Photodynamic therapy
MB-49: Murine bladder 49
MB-49-luc: MB-49 luciferase-expressing
PBS: Phosphate-buffered saline
IHC: Immunohistochemistry
HIER: Heat-induced epitope retrieval
H&E: Hematoxylin and eosin
OS: Overall survival
MFS: Metastasis-free survival
PFS: Progression-free survival
VTPS: SbVTP and surgical resection
IFN: Intracellular interferon
APCs: Antigen-presenting cells
