# Supplementary material for: Neoadjuvant vascular-targeted photodynamic therapy improves survival and reduces recurrence and progression in a mouse model of urothelial cancer
Source: Sci Rep. 2021 Mar 1;11:4842. doi: 10.1038/s41598-021-84184-y (PMC7921650; doi:10.1038/s41598-021-84184-y)
Supplement: Supplementary file 1 — Supplementary Information. [file 41598_2021_84184_MOESM1_ESM.docx]

**Supplementary material**

**Neoadjuvant Vascular-Targeted Photodynamic Therapy Improves Survival and Reduces Recurrence and Progression in a Mouse Model of Urothelial Cancer**

Barak Rosenzweig^1,2^, Renato B. Corradi^3^, Sadna Budhu^4^, Ricardo Alvim^3^, Pedro Recabal^1^, Stephen La Rosa^3^, Alex Somma^3^, Sebastien Monette^5,6^, Avigdor Scherz^7^, Kwanghee Kim^3^ and Jonathan A. Coleman^1,6^

^1^ Department of Surgery, Urology Service, Memorial Sloan Kettering Cancer Center, New York, NY

^2^ Department of Urology. The Chaim Sheba Medical Center, Ramat Gan, Israel, affiliated with the Sackler School of Medicine,

^3^ Department of Surgery, Sloan-Kettering Institute, Memorial Sloan Kettering Cancer Center, New York, NY

^4^ Immunology Program, The Jedd Wolchok Lab, Memorial Sloan Kettering Cancer Center, New York, NY

^5^ Laboratory of Comparative Pathology, Memorial Sloan Kettering Cancer Center, New York, NY

^6^ Weill Cornell Medical College, New York, NY

^7^ Department of Plant Sciences, Weizmann Institute of Science, Rehovot, Israel

**Supplementary Table 1**. Tumor and lung fluorescence signal (photons/seconds/cm^2^/steradian) by study group

| **Fluorescence signal by group** |  | **Control** | **Early surgery** | **Early sbVTP** | **Late surgery** | **Late sbVTP** |
| --- | --- | --- | --- | --- | --- | --- |
| **Tumor** | Mean | 8.97E+06 | 1.26E+03 | 2.69E+06 | 1.03E+05 | 4.47E+06 |
|  | standard deviation | 8.01E+06 | 1.85E+03 | 4.50E+06 | 3.37E+05 | 7.46E+06 |
|  | 95% confidence interval | 4.01E to 1.39E+07 | 1.68E+02 to 2.35E+03 | 2.52E+04 to 5.35E+06 | 0 to 3.02E+05 | 8.88E+06 |
| **Lungs** | Mean | 3.71E+04 | 3.35E+02 | 2.53E+03 | 5.51E+03 | 1.14E+04 |
|  | standard deviation | 6.74E+04 | 4.45E+01 | 5.59E+03 | 8.80E+03 | 2.83E+04 |
|  | 95% confidence interval | 0 to 7.89E+04 | 3.09E+02 to 3.61E+02 | 0 to 5.83E+03 | 3.11E+02 to 1.07E+04 | 0 to 2.81E+04 |


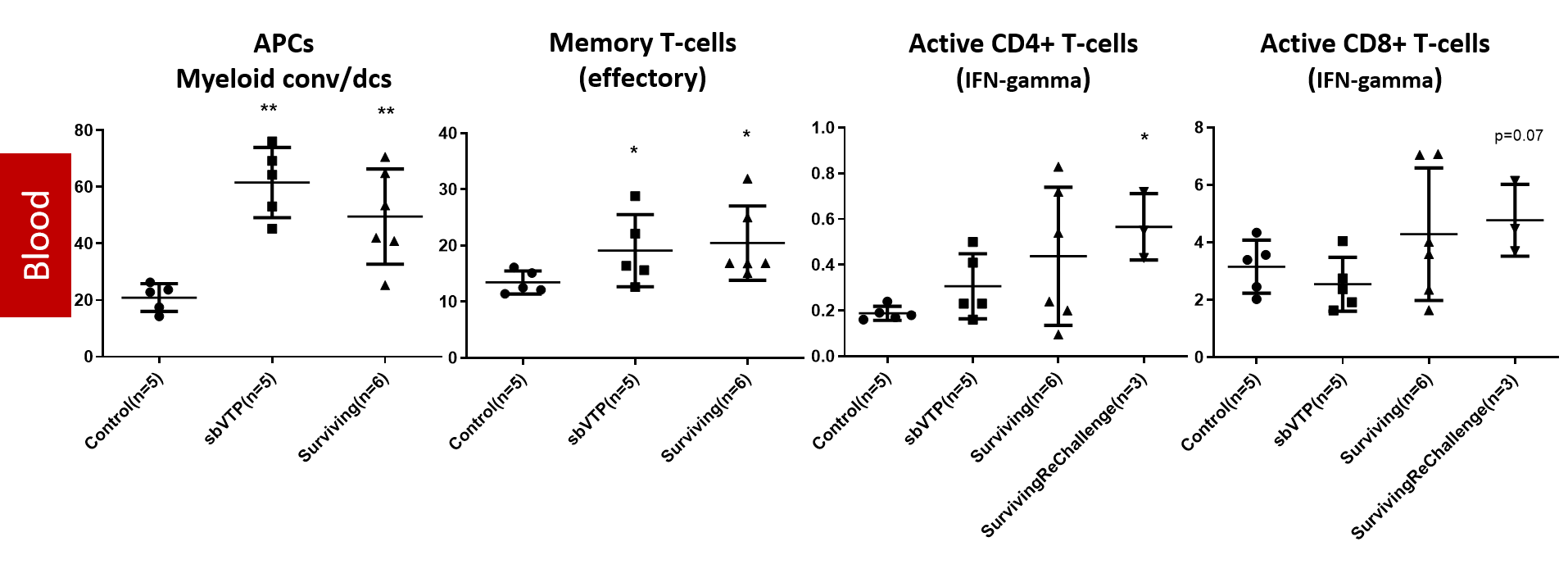


**Supplementary Figure 1.** Blood flow data: cells’ sub-population percentage. Left to right: first column represents antigen-presenting cells (APCs), second column represents memory T-cells (effector), third and fourth columns represents active CD4+ and CD8+ T-cells, respectively, as indicated by IFN-gamma positivity. CD45+, CD4+, Foxp3- were used to evaluate CD4 T effector cells. CD62L+CD44+ were used to evaluate central memory T cells and CD62L-CD44+ to evaluate effector or effector memory T cells. APCs = antigen-presenting cells; conv = conventional; dcs = dendritic cells; IFN = interferon; sbVTP = sub-ablative vascular-targeted photodynamic therapy; surviving = surviving animals; surviving rechallenge= surviving animals rechallenged with tumor cells. *, *P* ≤ 0.05; **, *P* < 0.01, compared to control.
